# Purpura Rheumatica

**Published:** 1913-01

**Authors:** 


PURPURA RHEUMATICA—Gonalons, (*La Semana Medica,* Aug. 15, 1912) reports a case which appeared following a typic attack of acute articular rheumatism and two months after all rheumatic symptoms had disappeared. The eruption appeared on three separate occasions, lasting two or three days, hemorrhagic, confluent and accompanied by bilious vomiting, joint pains and headache. Later, hemorrhages from the bowel set in. Amenorrhea existed for three months previous to the appearance of the eruption and continued for more than a month afterward when a short metrorhagia supervened. The bleeding was controlled by horse serum and the chloride of calcium.

